# Timing Is Everything, Right? Meal Impact on Circadian Related Health

**DOI:** 10.1210/clinem/dgaa533

**Published:** 2020-11-03

**Authors:** Steven K Malin

**Affiliations:** 1 Department of Kinesiology & Health, Rutgers University, New Brunswick, NJ; 2 Division of Endocrinology, Metabolism & Nutrition, Rutgers University, New Brunswick, NJ; 3 Center for Nutrition, Exercise & Metabolism, Rutgers University, New Brunswick, NJ; 4 Institute of Translational Medicine & Science, Rutgers University, New Brunswick, NJ

## Invited Commentary

Although there has been much debate in recent years on the optimal dietary macronutrient composition for combating obesity and promoting weight loss, most agree that total calories are of critical importance (1). However, times are evolving. In recent years chronobiology has emerged as a pivotal player in the genesis of metabolic disease (2, 3). It is now becoming clearer that food interacts with our circadian rhythm to impact physiologic processes. In fact, this is perhaps best exemplified by night shift workers having a high risk of obesity, type 2 diabetes, and cardiovascular disease (2–4). These observations have led many dietitians, exercise physiologists, basic scientists, and physicians to consider refocusing efforts on understanding both the efficacy of altering *when* people eat for health gain as well as elucidating the biology of the human circadian rhythm across tissues (eg, skeletal muscle, liver, brain, etc.). This, after all, is of public health and clinical relevance since adherence to conventional lifestyle modifications, including caloric restriction and physical activity, are challenging at the population level (5, 6). Accordingly, there is a strong need for contemporary recommendations that create sustained behavior change.

In this issue of the *Journal of Clinical Endocrinology & Metabolism,* novel work is presented examining the impact of isocaloric early versus late dinner on substrate metabolism, glucose regulation, and plasma lipids (7). Gu et al (7) tested the hypothesis that a late dinner would worsen metabolic health throughout sleep, and the following morning compared with a routine isocaloric dinner. To test this hypothesis, men and women ages 18–30 years and with obesity underwent a randomized crossover study whereby routine dinner was defined as 6:00 pm compared with a late dinner, defined as 10:00 pm. Meals (35% of total calories, 55% carbohydrate, 35% fat, and 15% protein) were provided under isocaloric conditions at set times throughout the day while people remained sedentary in the Clinical Research Unit. Venous blood draws for glucose, free fatty acids, triglycerides, insulin, cortisol, as well as palmitate isotopes, to determine fatty acid oxidation, were assessed hourly from 5:00 pm to 12:00 pm the next day. Sleep and chronotype were assessed by actigraphy and polysomnography as well as a questionnaire, respectively. The results showed that a late dinner induced greater glucose area under the curve following dinner and the next morning, whereas cortisol was higher over the averaged 20-hour period and insulin levels were statistically higher 2 hours after breakfast only. Late dinner also lowered circulating free fatty acids and delayed peaks in triglycerides following dinner into sleep, as well as reduced overall fatty acid oxidation. There were no apparent changes in sleep duration or patterns. While the effects of late dinner on pancreatic function (eg, C-peptides, GLP-1, glucagon, etc.) or muscle protein turnover (eg, growth hormone-related amino acid metabolism) were not a focus of the current work, these results collectively highlight that acute episodes of late dinner induces glucose intolerance and reduces fat utilization. Thus, further work is warranted to understand the pathologic underpinnings, as these data suggest that late dinner potentially raises obesity, type 2 diabetes, and/or metabolic syndrome risk.

Future work should consider the context of meal timing throughout the day for health. The work discussed herein (7) was performed in the evening when circadian biology demonstrates people are relatively glucose intolerant, insulin resistant, and have reduced endothelial function compared with the morning (2, 3). But how would the effects of a late dinner on metabolism differ if morning or lunch meals were adjusted away from routine times and in line with dinner? To that end, how would the health outcome responses to late dinner differ under conditions of energy deficit compared with energy balance/surplus? This is likely important to consider given modern-day interest in time restriction feeding or intermittent fasting as a weight loss approach as well as the impact of energy availability as a mechanism for metabolic adaptation. Another consideration is that the food (ie, 35% of total calorie needs) provided by Gu et al (7) was relatively mixed in macronutrients. It is unclear though if altering the macronutrient content (low vs high carbohydrate/protein or processed vs unprocessed) before or at dinner would make a difference to health outcomes based on meal timing and circadian biology. Alternatively, perhaps the individuals under treatment ought to be highlighted too. Gu et al (7) identified that people with obesity who were categorized as habitually early sleepers had more prominent responses to the late dinner than late sleepers. This is an intriguing finding since it points towards some individuals’ sleep habits as being more susceptible to food interacting with circadian rhythm than others. Further, it is worth recognizing that these participants were relatively sedentary (7) and that the consideration of meals in proximity to human movement may be of importance for thwarting chronic disease (8). Interestingly, athletes are often encouraged to consume certain foods at given times in the day (eg, pre-/postexercise or before sleep) to maximize tissue repair, increase protein synthesis, and elevate glycogen deposition. However, despite recommendations by some to consume meals later in the day as a strategy to ensure proper caloric/macronutrient intake, disease risk remains low in people with high aerobic/muscular fitness. This raises the possibility that fitness status may alter the responses to late meal consumption during the wake period. Therefore, as work in this area emerges, attention may need to be paid to the consideration of meal timing recommendations and their need to shift as one goes from a sedentary lifestyle to an active lifestyle (or vice a versa) to optimize and sustain health. In either case, one benefit of recommending to alter or confine meal timing at least early on in a health program is that it focuses on *when* and not *what* people should eat. This approach may prove advantageous in reducing perceptions of dietary restrictions being difficult (eg, hunger). Taken together, the current work adds to a growing body of literature that food interacts with our circadian rhythm to impact disease risk. Whether meal timing recommendations affect lifestyle adherence as well as enhances individual well-being awaits to be seen.

## Data Availability

Data sharing is not applicable to this article, as no datasets were generated or analyzed during the current study.

## Additional Information

***Disclosure Summary:*** The author has nothing to disclose.
